# Conceptual design of a generic data harmonization process for OMOP common data model

**DOI:** 10.1186/s12911-024-02458-7

**Published:** 2024-02-26

**Authors:** Elisa Henke, Michele Zoch, Yuan Peng, Ines Reinecke, Martin Sedlmayr, Franziska Bathelt

**Affiliations:** 1 Institute for Medical Informatics and Biometry, Carl Gustav Carus Faculty of Medicine, Technische Universität Dresden, 01307 Dresden, Germany; 2 Data Integration Center, Center for Medical Informatics, University Hospital Carl Gustav Carus Dresden, 01307 Dresden, Germany; 3 Thiem-Research GmbH, 03048 Cottbus, Germany

**Keywords:** OMOP, OHDSI, Interoperability, Data harmonization, Clinical data, Claims data

## Abstract

**Background:**

To gain insight into the real-life care of patients in the healthcare system, data from hospital information systems and insurance systems are required. Consequently, linking clinical data with claims data is necessary. To ensure their syntactic and semantic interoperability, the Observational Medical Outcomes Partnership (OMOP) Common Data Model (CDM) from the Observational Health Data Sciences and Informatics (OHDSI) community was chosen. However, there is no detailed guide that would allow researchers to follow a generic process for data harmonization, i.e. the transformation of local source data into the standardized OMOP CDM format. Thus, the aim of this paper is to conceptualize a generic data harmonization process for OMOP CDM.

**Methods:**

For this purpose, we conducted a literature review focusing on publications that address the harmonization of clinical or claims data in OMOP CDM. Subsequently, the process steps used and their chronological order as well as applied OHDSI tools were extracted for each included publication. The results were then compared to derive a generic sequence of the process steps.

**Results:**

From 23 publications included, a generic data harmonization process for OMOP CDM was conceptualized, consisting of nine process steps: dataset specification, data profiling, vocabulary identification, coverage analysis of vocabularies, semantic mapping, structural mapping, extract-transform-load-process, qualitative and quantitative data quality analysis. Furthermore, we identified seven OHDSI tools which supported five of the process steps.

**Conclusions:**

The generic data harmonization process can be used as a step-by-step guide to assist other researchers in harmonizing source data in OMOP CDM.

**Supplementary Information:**

The online version contains supplementary material available at 10.1186/s12911-024-02458-7.

## Background

The use of real-world data for research is becoming increasingly important in order to gain insights into the real-life care of patients in the healthcare system and, on this basis, to gain new knowledge for the diagnosis, treatment and prevention of diseases. To promote digitization in medicine for the areas of care and research in Germany, the Medical Informatics Initiative (MII) has been funded since 2018 by the German Federal Ministry for Education and Research (BMBF) [1]. The aim of the MII is to link data from patient care by providing digital infrastructures for the integration and harmonization of health data for research purposes. However, the developments of the infrastructures are currently focused on patient data of German university hospitals. Therefore, research in the MII is limited to clinical data from patients during hospitalization. The medical care of patients in university hospitals, in contrast, affects only a small percentage, since patients are usually hospitalized only when they already have a severe disease.

Green et al. [2] pointed out that a far greater number of patients are treated outside of the hospital. In comparison to inpatient data, outpatient data provide a more comprehensive overview of patients’ medical histories. A relevant data source for outpatient data is claims data from the statutory health insurance funds in Germany. By linking claims data across institutions and sectors on a person-specific basis, longitudinal analyses of treatment histories can be realized. However, due to their billing focus, claims data lack depth of content, so that information on, for example, diagnostic and laboratory data is not included. In order to integrate both detailed information on the respective inpatient stay of patients as well as the insured person-related course perspective for research with real-world data, the combination of clinical data with claims data is necessary.

To exploit the potential of linking clinical data with claims data, it is important to ensure the syntactic and semantic interoperability of both data sets. Syntactic interoperability focuses on the definition of standardized data formats and information models, while semantic interoperability aims to achieve a uniform understanding of information models and terminology content across systems [3]. Achieving syntactic and semantic interoperability requires data harmonization, i.e. transforming local source data into a standardized format [4]. For the unified representation of heterogeneous data sets, so-called standardized common data models (CDMs) are developed. In the last years, the Observational Medical Outcomes Partnership (OMOP) CDM of the Observational Health Data Sciences and Informatics (OHDSI) gained significant relevance for research with real-world data [5–9].

The main challenge for researchers is the harmonization of national and institution-specific terminologies, formats and structures into the standardized format of OMOP CDM. For this purpose, OHDSI provides tools and introduces four major steps that should help to harmonize source data in OMOP CDM: Design the Extract-Transform-Load (ETL), Create the Code Mappings, Implement the ETL, Quality Control [10] (pp. 75–94). However, our own experience in harmonizing clinical data of the MII given in the Fast Healthcare Interoperability Resources (FHIR) format to OMOP CDM [11] has shown that these steps are not detailed enough. The literature demonstrates that many researchers are concerned with harmonization of source data in OMOP CDM [6]. Nevertheless, there is no detailed guidance that would allow researchers to follow a generic process when transforming source data to OMOP CDM, which is independent of type of source data used. A generic process is necessary to ensure the reusability of methods and tools as well as the reproducibility and comparability of results.

Prior to the practical harmonization of German claims data in OMOP CDM, we first investigated how such a generic process would look like in theory. Thus, the aim of this paper is to conceptualize a generic data harmonization process for OMOP CDM that is applicable for clinical data and claims data. In this context, we focus on the following three research questions:

1. Which process steps need to be performed when harmonizing clinical data or claims data in OMOP CDM?
2. What OHDSI tools were used by researchers to support the harmonization of clinical data or claims data in OMOP CDM?
3. What sequence of identified process steps should be followed?

## Methods

### Literature review

#### Paper identification

To obtain a clearer perspective of the state of the art of methodological processes for data harmonization in OMOP CDM, we conducted a literature review on August 3, 2023. Our literature search included publications published in English between 2018 and 2023, focusing on the harmonization of clinical data or claims data in OMOP CDM. Table 1 provides an overview of the search terms used in the literature databases PubMed and Web of Science.

The resulting publications were imported into the reference management software Zotero [12]. Afterwards, duplicates were removed using Zotero’s built-in duplicate detection feature.

**Table 1:** Search terms used for the literature search in PubMed and Web of Science

Database	Search String
PubMed	((OMOP[Title/Abstract]) OR (OHDSI[Title/Abstract])) AND ((claims data[Title/Abstract]) OR (clinical data[Title/Abstract]))
Web of Science	((TI=(OMOP) OR TI=(OHDSI)) AND (TI=(claims data) OR TI=(clinical data))) OR ((AB=(OMOP) OR AB=(OHDSI)) AND (AB=(claims data) OR AB=(clinical data)))

#### Paper exclusion

The process of paper exclusion consisted of a Title-Abstract-Screening and Full-Text-Screening performed by three reviewers (EH, FB, MZ). For this purpose, nine exclusion criteria (Table 2) were defined to categorize the excluded publications.

**Table 2:** Definition of exclusion criteria following to Reinecke et al. [6]

Criterion	Description of criterion
no “OMOP” or “OHDSI”	Publication does not mention “OMOP” or “OHDSI”
	Publication uses “OMOP” or “OHDSI” with other meanings
mentioned	Publication only mentions “OMOP” or “OHDSI”
evaluated	Publication focuses on the evaluation of OMOP
vocabulary	Publication focuses on vocabularies and their mapping in OMOP or use of OMOP vocabularies
extension	Publication focuses on an extension of OMOP or OHDSI tools
usage	Publication focuses on the use of OMOP, e.g. for studies, data quality analyses, development of tools or frameworks (e.g. patient level prediction)
no full text	Publication is not available as full text
foreign language	Publication is written in other languages than English
wrong type of source data	Publication focuses on types of source data other than clinical data or claims data

Next, all three reviewers performed the Title-Abstract-Screening for 20% of the publications found. Conflicts were then discussed and resolved. Afterwards, we utilized the kappa statistic to test the interrater reliability [13]. For this purpose, we used the Fleiss method [13] in the KappaM function of the R library *DescTools* [14]. For the analysis, an error probability of 5% was set. Depending on the result of the kappa value, we chose one of the two defined options for the further procedure of paper exclusion:

- Option 1: For a kappa value greater than 0.6 (substantial to almost perfect agreement (interpretation according to Fleiss [13])), the Title-Abstract-Screening for the remaining 80% of the publications found and afterwards, the Full-Text-Screening for the included publications should be divided as follows: (1) reviewer 1 (EH) should screen all publications; (2) reviewer 2 (FB) all included publications and (3) reviewer 3 (MZ) all excluded publications.
- Option 2: If the kappa value is less than or equal to 0.6 (poor to moderate agreement (interpretation according to Fleiss [13])), the remaining 80% of the publications and the full texts had to be screened by all three reviewers.

After both, Title-Abstract-Screening and Full-Text-Screening, conflicts between the reviewers were discussed and resolved.

#### Data extraction

After the paper exclusion process, we focused on data extraction from the included publications. The data extraction was performed by reviewer 1 (EH) and subsequently verified by reviewer 2 (FB). The data extraction process consisted of three iterations. The first iteration focused on extracting the process steps and OHDSI tools from the publications used to harmonize the source data in OMOP CDM. In addition, the specification of the type of source data (clinical data and/or claims data) used for data harmonization and the countries from which the data was originated were documented for each publication.

In a second iteration, for each publication, we checked which of the extracted process steps and OHDSI tools were applied during the data harmonization in OMOP CDM. For this purpose, we created a matrix. The columns of the matrix represented the extracted process steps and OHDSI tools, while the rows represented the included publications. Within the matrix, we used crosses to indicate when a process step or OHDSI tool was mentioned in the corresponding publication.

In the final third iteration, the focus was on identifying the chronological order of the applied process steps per publication. For this purpose, we replaced the crosses of the process steps in the matrix with ascending numbers. Afterwards, the distribution of the given numberings per process step was calculated and the most frequent number was highlighted. This approach was performed for each of the publications that (a) used clinical data as source data or (b) used claims data as source data and (c) for all publications regardless of the type of source data. Publications that used both, clinical data and claims data, were categorized into all three groups.

### Derivation of a generic sequence of process steps

In order to be able to apply the process steps to the harmonization of source data, it was necessary to establish a chronological classification of the process steps in an overall process. For this purpose, we compared the most frequent number(s) per process step. Our comparison started with the process step with the lowest number. Thereafter, we compared it to the number of the next process step. If the subsequent process step had a higher numbering (e.g. *1* vs. *2*), both process steps remained in their position. In the case of two identical numberings (e.g. *1*), the higher sum of the percentage of the most frequent numbering and the percentages of the preceding numberings decided the position in the comparison and all subsequent numberings were increased by the value *1*. This method of comparing predecessor and successor numbers was used until a unique numbering and thus positioning in the overall process could be defined. The derivation of the sequence of process steps was done separately for groups a)-c) defined in Sect. Data extraction. Finally, the results of the three groups were compared with regard to their agreement to derive a generic sequence for the data harmonization process.

## Results

### Flow diagram of the literature review

Based on the search terms, 162 publications (PubMed: 55, Web of Science: 107) were found. After removing duplicates in Zotero (34 publications), 128 publications remained for Title-Abstract-Screening. During the Title-Abstract-Screening, all three reviewers initially reviewed 20% (26/128) of the publications. Then we calculated the kappa value to check reviewer agreement. The kappa statistic resulted in a value of κ = 0.764 (substantial agreement). Since this value was greater than 0.6, we chose Option 1 (see Sect. Paper exclusion) for the further paper exclusion process. After Title-Abstract-Screening, 85 publications were excluded according to the definition of the exclusion criteria in Table 2. In the subsequent screening of the full texts of 43 publications, we further excluded 20 publications. Finally, 23 publications were included for data extraction.

**Fig. 1:**
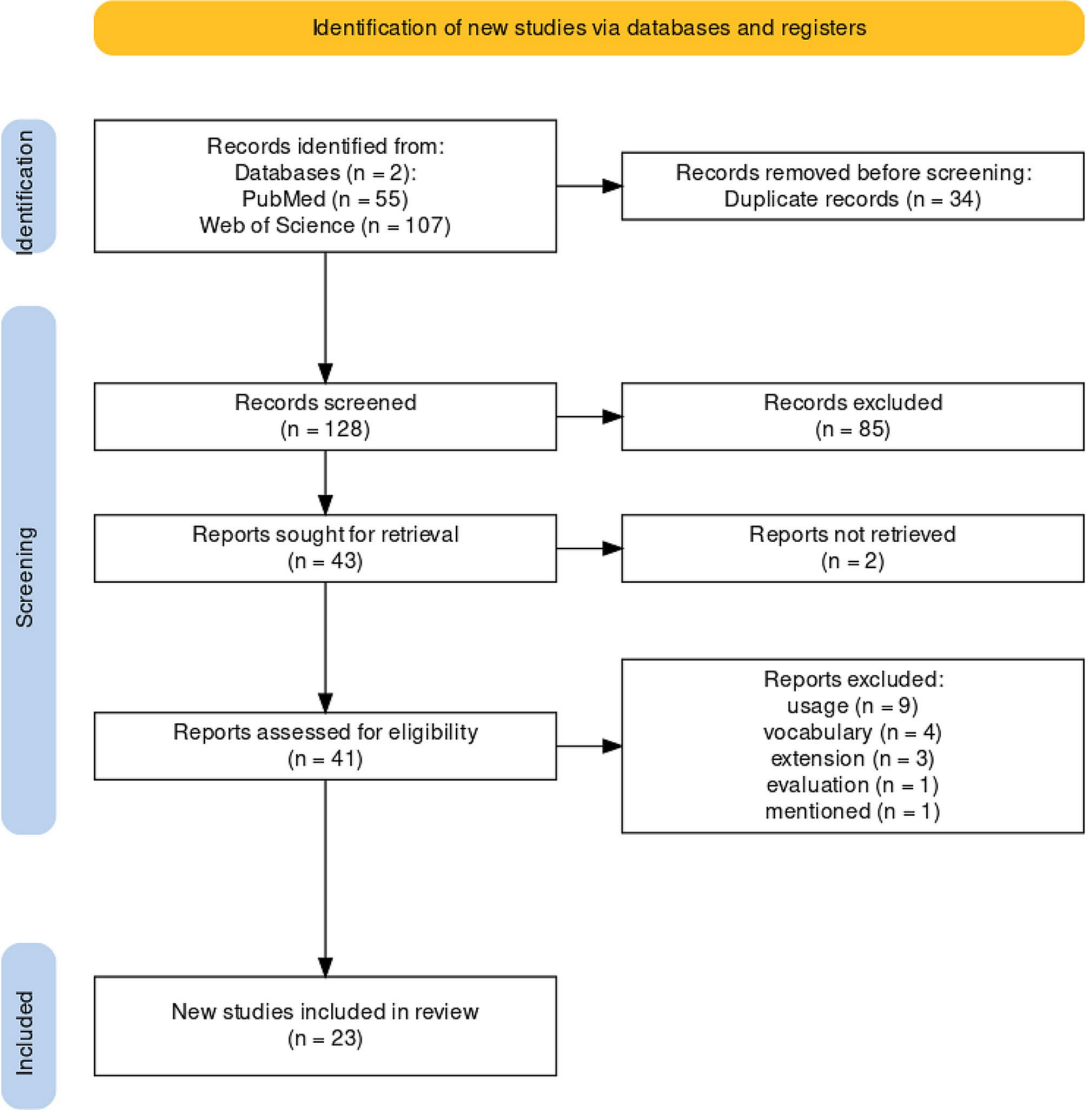
PRISMA flow diagram according to [15]

Figure 1 summarizes the process of the literature search, the subsequent screening of publications for inclusion and the remaining publications for the data extraction as PRISMA (Preferred Reporting Items for Systematic reviews and Meta-Analyses) flow diagram [16]. A detailed overview of the results of the literature search, including the data extraction matrix can be found in the Additional file 1.

### Process steps and OHDSI tools

The 23 reviewed publications allowed us to extract a methodological process for data harmonization in OMOP CDM [17–39]. Clinical data were used in 18 of 23 publications (78%) [17, 18, 20–29, 32–34, 36, 37, 39], claims data were used in 3 of 23 publications (13%) [30, 31, 35], and both types of data sources were used in 2 of 23 publications (9%) [19, 38] as source data for data harmonization in OMOP CDM. Clinical data originated from Belgium, Brazil, China, Denmark, Estonia, France, Germany, Italy, the Netherlands, Portugal, Serbia, Singapore, South Korea, Spain, Turkey, the United Kingdom and the United States. Claims data were used from Austria, France, and the United States (see Additional file 1).

The methodological process extracted from the publications consists of the nine process steps. Furthermore, we identified seven OHDSI tools which were used in the literature to support the harmonization of clinical data or claims data in OMOP CDM. In the following, the nine process steps are explained (in alphabetical order) and the seven OHDSI tools are assigned to them:

- In order to assess the extent to which the vocabularies found in the source data can already be mapped in OMOP CDM, a **coverage analysis of the vocabularies** of the source data is performed. The analysis helps to identify weaknesses (e.g. missing vocabularies) that would limit a full harmonization of the source data. Through this process step, Rinner et al. found that the Anatomical Therapeutic Chemical Classification and the International Statistical Classification of Diseases and Related Health Problems Tenth Revision existed in OMOP CDM, while the Austrian pharmaceutical registration number and a catalogue of medical services were missing [30].
- To get an overview of the source data including their structure, formats and unique values, a **data profiling** is performed. For this purpose, OHDSI provides the tool WhiteRabbit [40] to analyze the source data.
- **Dataset specification** refers to the definition of the scope of the source data for a specific use case. This is usually done by expert teams with clinical expertise. As a result, transformation to OMOP CDM is only performed on source data that is relevant to answering a specific research question.
- The technical transformation of the source data into OMOP CDM is realized through the implementation of **ETL-processes**. ETL-processes enable the reading of source data (Extract), the practical implementation of semantic and structural mapping (Transform), and the final writing of OMOP-compliant source data to the target database (Load).
- The **qualitative data quality analysis** examines, in particular, the plausibility, conformity and completeness of the source data in OMOP CDM (according to Kahn et al. [41]). With the Automated Characterization of Health Information at Large-scale Longitudinal Evidence Systems (Achilles) [42] and the Data Quality Dashboard (DQD) [43], two OHDSI tools exist that perform data quality checks on the transformed source data in OMOP CDM. The use of both OHDSI tools was described by Papez et al., who further investigated failed checks during multiple iterations and thus increased the qualitative data quality of their transformed data [23].
- The **quantitative data quality analysis** checks whether the number of data in the source matches the number of records in OMOP CDM. For this purpose, the OHDSI tool Atlas [44] can be used to define cohorts based on OMOP CDM. The number of cohorts can then be compared with cohorts based on source data. Another example is provided by Yu et al., who compared record counts per variable, over time and null values for all OMOP CDM Table [20]. Haberson et al. focused on calculating and comparing descriptive statistical indicators (e.g. median value for age or number of hospitalizations per person) for the source and transformed data [31].
- **Semantic mapping** refers to the mapping of local vocabularies to the standardized vocabulary of OMOP CDM [10] (pp. 55–74). This step is necessary to be able to uniquely identify source values by concepts in OMOP CDM and to transfer source values to standard concepts to enable research in an international context. The standardized vocabulary of OMOP CDM is provided by the OHDSI vocabulary repository Athena [45]. Furthermore, the OHDSI tool Usagi [46] supports researchers in semantic mapping of source values to OMOP CDM concepts. The publication of Ji et al. describes the semantic mapping of the Korean Standard Classification of Diseases 7 to Systematized Nomenclature of Human and Veterinary Medicine Clinical Terms (SNOMED-CT) or the Korean Drug to RxNorm or RxNorm extension [27].
- The focus of the **structural mapping** is the conversion of the format of the source data into the standardized data model of OMOP CDM [10] (pp. 31–54). The structural mapping can be done by using the OHDSI tool RabbitInAHat [40].
- **Vocabulary identification** focuses on providing a comprehensive compilation of the vocabularies found in the source data, including their scope of application. For example, Papez et al. identified three vocabularies in UK primary care data (SNOMED-CT, Clinical Terms Version 3, Dictionary of Medicines and Devices), two vocabularies in hospital care data and mortality data (International Statistical Classification of Diseases and Related Health Problems Tenth Revision and Ninth Revision), one vocabulary for cancer registry data (International Classification of Diseases for Oncology, Third Edition) and two vocabularies in procedure data (OPCS Classification of Interventions and Procedures Version 3 and Version 4) [23].

#### Frequency

**Fig. 2:**
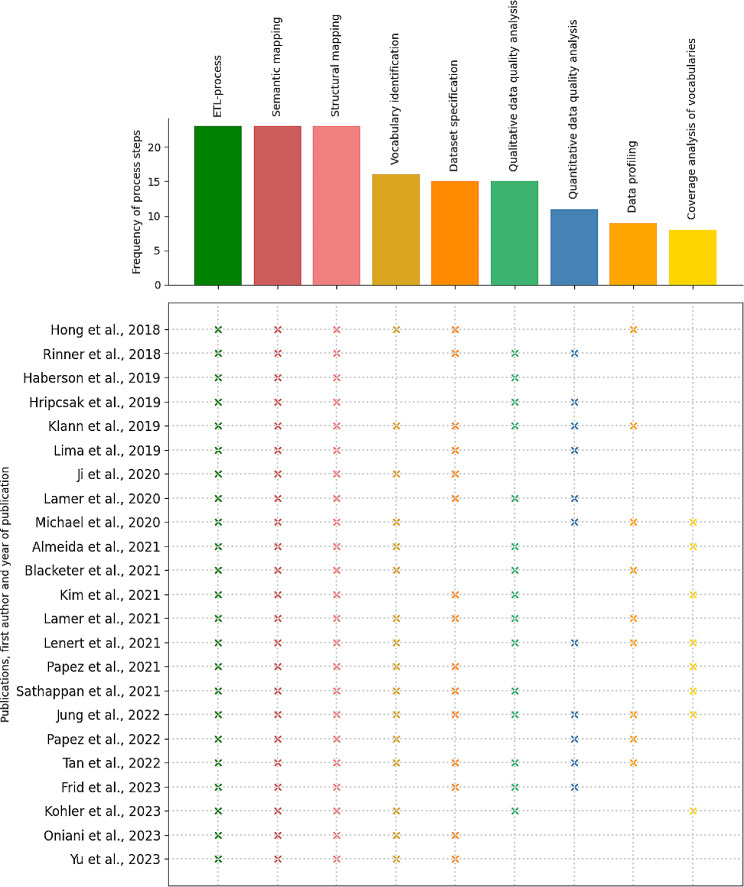
Frequency distribution of the extracted process steps and their assignment to the included publications

The frequency of the process step occurrence in the literature is shown in Fig. 2 in descending order. All publications included the implementation of ETL processes, semantic mapping and structural mapping. Furthermore, understanding the source data and mapping it to OMOP CDM was also important during data harmonization. This applies in particular to vocabulary identification (70%: 16/23), dataset specification (65%: 15/23), data profiling (39%: 9/23) and coverage analysis of vocabularies (35%: 8/23). About half of the publications dealt with qualitative (65%: 15/23) and quantitative data quality analyses (48%: 11/23) after successful transformation of the source data into OMOP CDM.

Furthermore, we also determined the quantitative occurrence of the seven identified OHDSI tools in the 23 included publications. The results showed that Athena and Achilles were used most frequently with 43% (10/23), followed by WhiteRabbit, RabbitInAHat and Usagi with 26% (6/23) each. The Data Quality Dashboard and Atlas were the least used in the literature with only 22% (5/23) each.

#### Chronological order

In order to define a generic sequence for the extracted process steps for data harmonization in OMOP CDM, the chronological order of the process steps was focused during the third iteration of data extraction. The chronological order of the process steps per publication can be found in the Additional file 1.

**Fig. 3:**
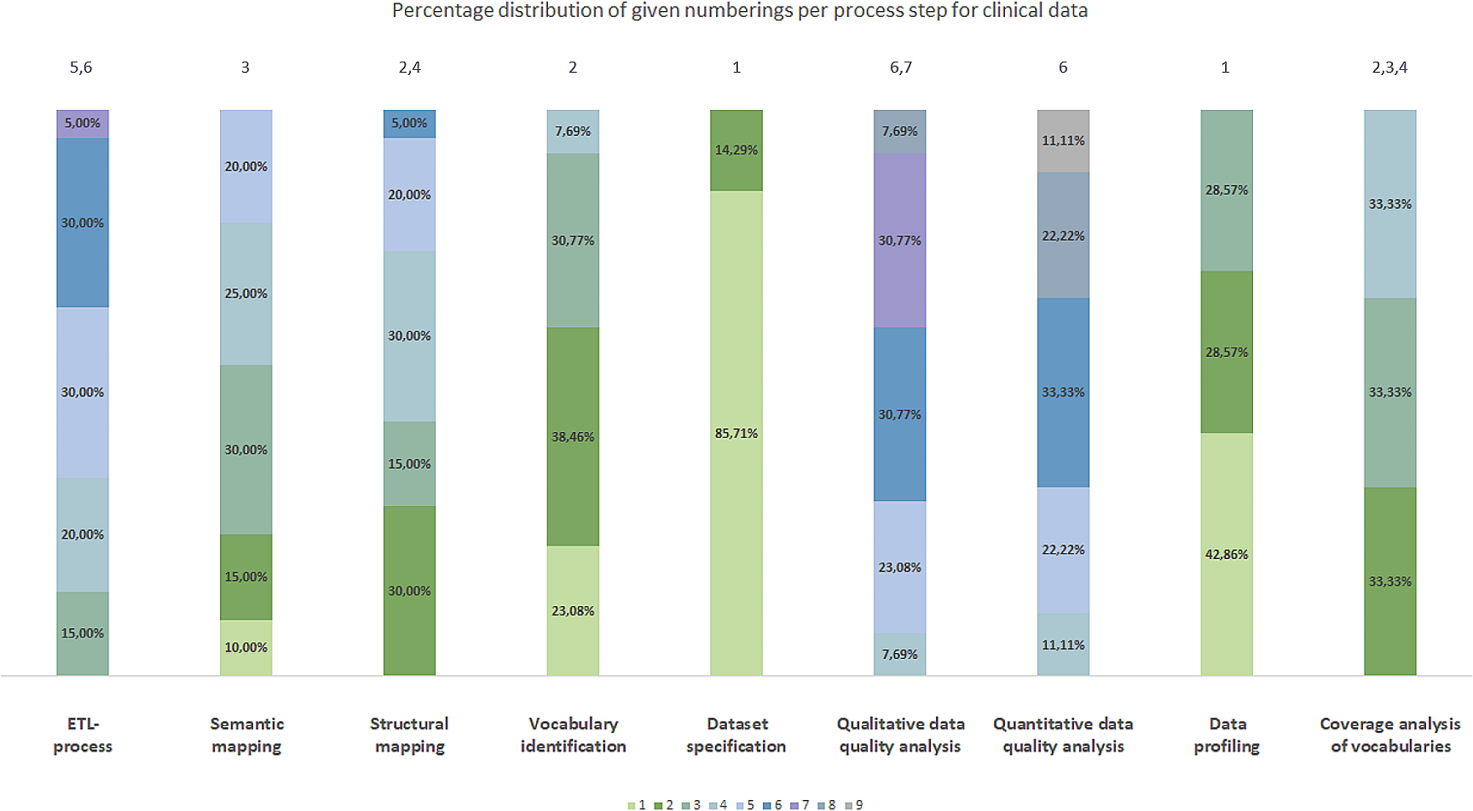
Percentage distribution of given numberings per process step for clinical data (group a))

**Fig. 4:**
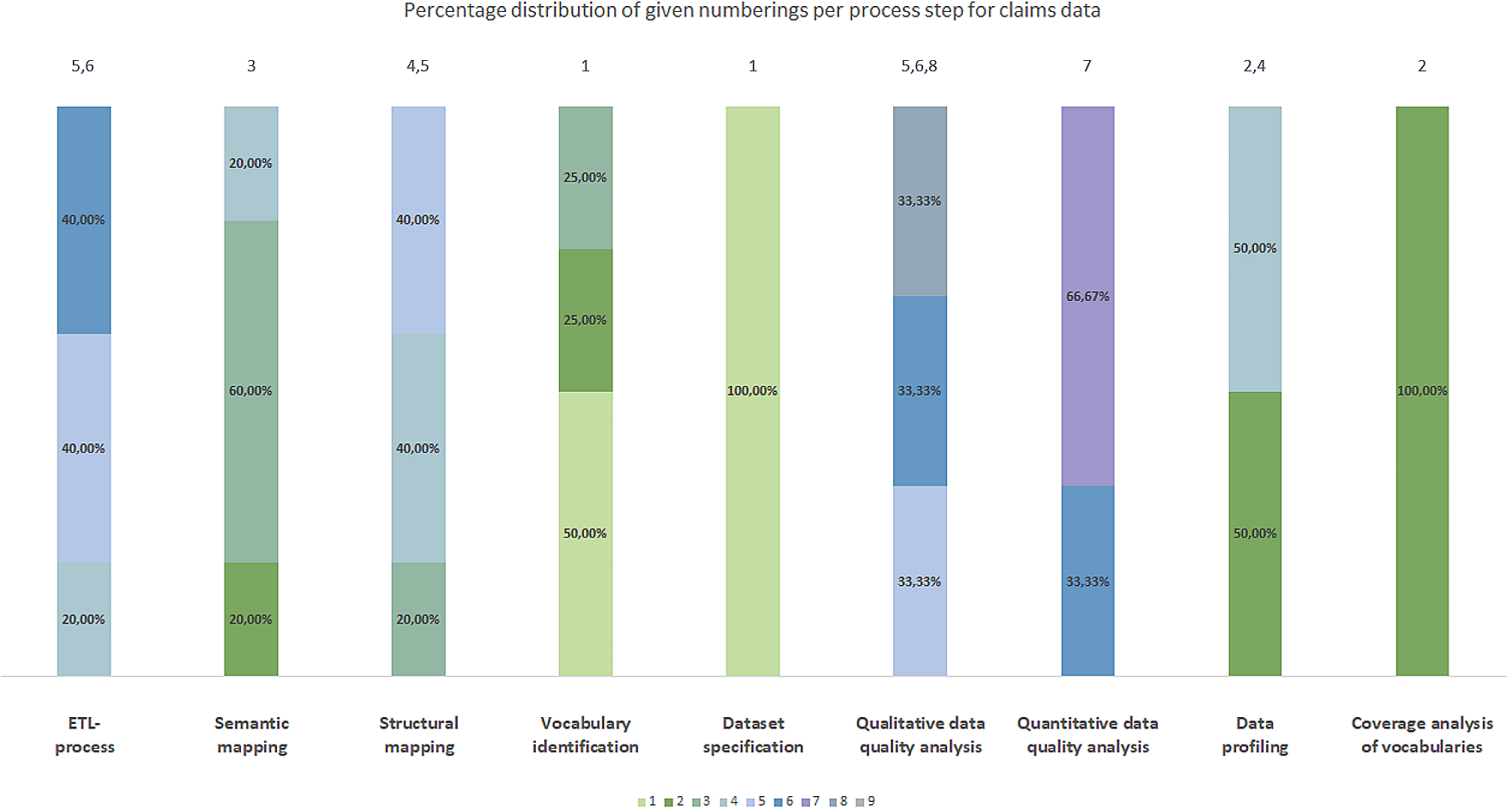
Percentage distribution of given numberings per process step for claims data (group b))

**Fig. 5:**
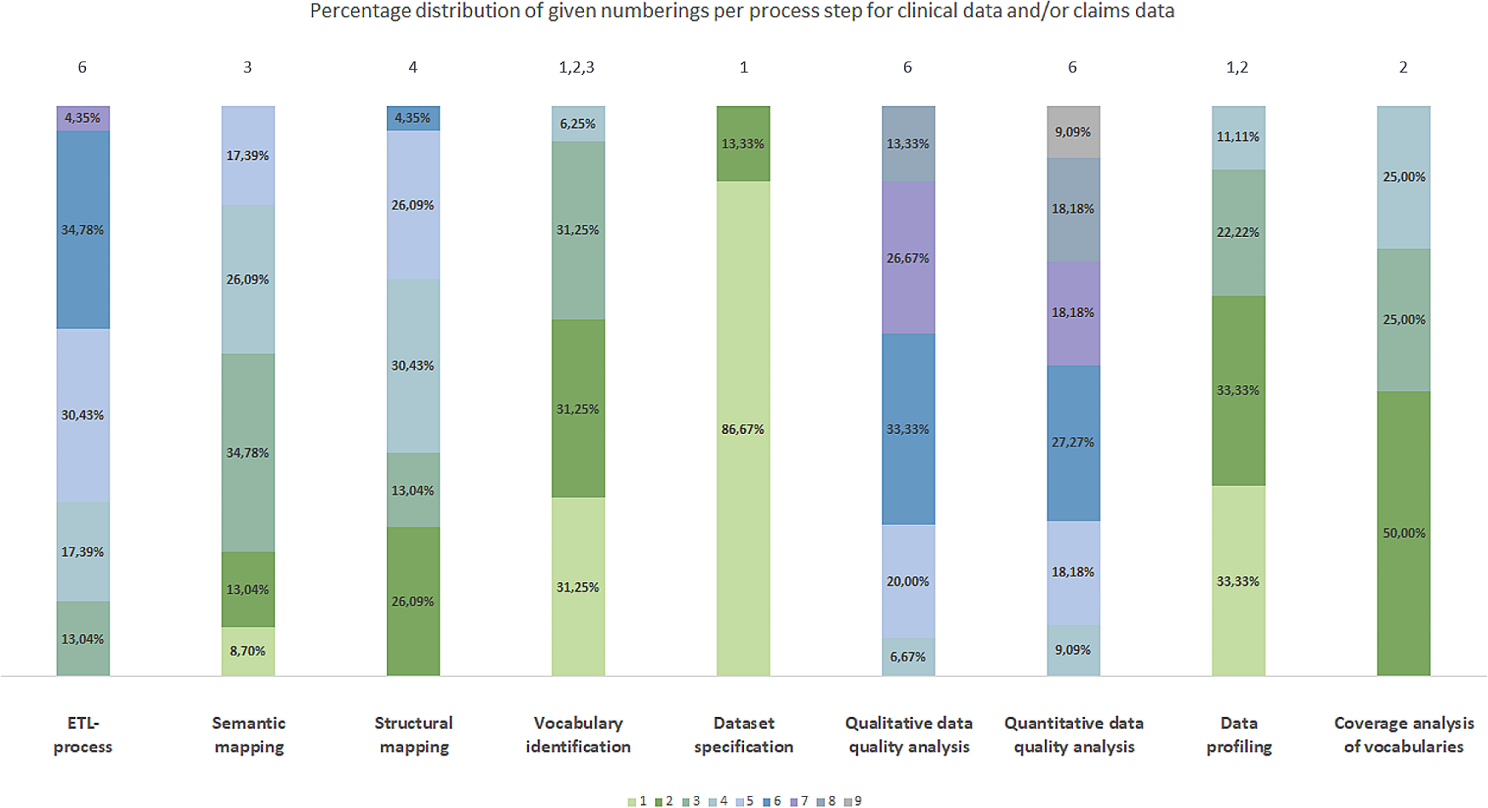
Percentage distribution of given numberings per process step for clinical data and/or claims data (group c))

To calculate the percentage distribution of the given numberings per process step, we assigned the included publications to the three groups a)-c). This resulted in the following number of publications per group: group a): 20 publications, group b): five publications and group c): 23 publications. Figures 3, 4 and 5 represent the percentage distribution as well as the indication of the most frequent numbering(s) for the groups a)-c).

The three diagrams show that the numbers *1* to *9* can occur several times as the most frequent numbering per group (e.g. number *1* in Fig. 3). Therefore, it was not yet possible to obtain a clear positioning of the identified process steps in an overall data harmonization process for each group.

### Generic data harmonization process

By comparing the most frequent number(s) per process step according to our approach in Sect. Derivation of a generic sequence of process steps, we were able to determine the chronological order of the data harmonization process for the groups a)-c). Table 3 summarizes the results separated by the type of source data. The results show, that there are similarities (highlighted using bold text) and deviations of the chronological order of the process steps between the three groups. All three groups match completely for process numbers *1* (dataset specification) and *7* (ETL-process). For the remaining process numbers, there are deviations between the three groups. This applies to the exchange of structural mapping and semantic mapping or quantitative and qualitative data quality analyses for clinical data, as well as for the vocabulary identification, coverage analysis of vocabularies and data profiling for claims data. Nevertheless, two process steps per process number always match. This suggests that some process steps can also be interchanged with each other and the goal of harmonizing source data in OMOP has nevertheless been achieved by other researchers.

**Table 3:** Chronological order of process steps separated by type of source data

Process number/ Type of source data	*1*	*2*	*3*	*4*	*5*	*6*	*7*	*8*	*9*
**Clinical data**	**Dataset specification**	**Data profiling**	**Vocabulary identification**	**Coverage analysis of vocabularies**	Structural mapping	Semantic mapping	**ETL-process**	Quantitative data quality analyses	Qualitative data quality analyses
**Claims data**	**Dataset specification**	Vocabulary identification	Coverage analysis of vocabularies	Data profiling	**Semantic mapping**	**Structural mapping**	**ETL-process**	**Qualitative data quality analyses**	**Quantitative data quality analyses**
**Clinical data and/or claims data**	**Dataset specification**	**Data profiling**	**Vocabulary identification**	**Coverage analysis of vocabularies**	**Semantic mapping**	**Structural mapping**	**ETL-process**	**Qualitative data quality analyses**	**Quantitative data quality analyses**

**Fig. 6:**
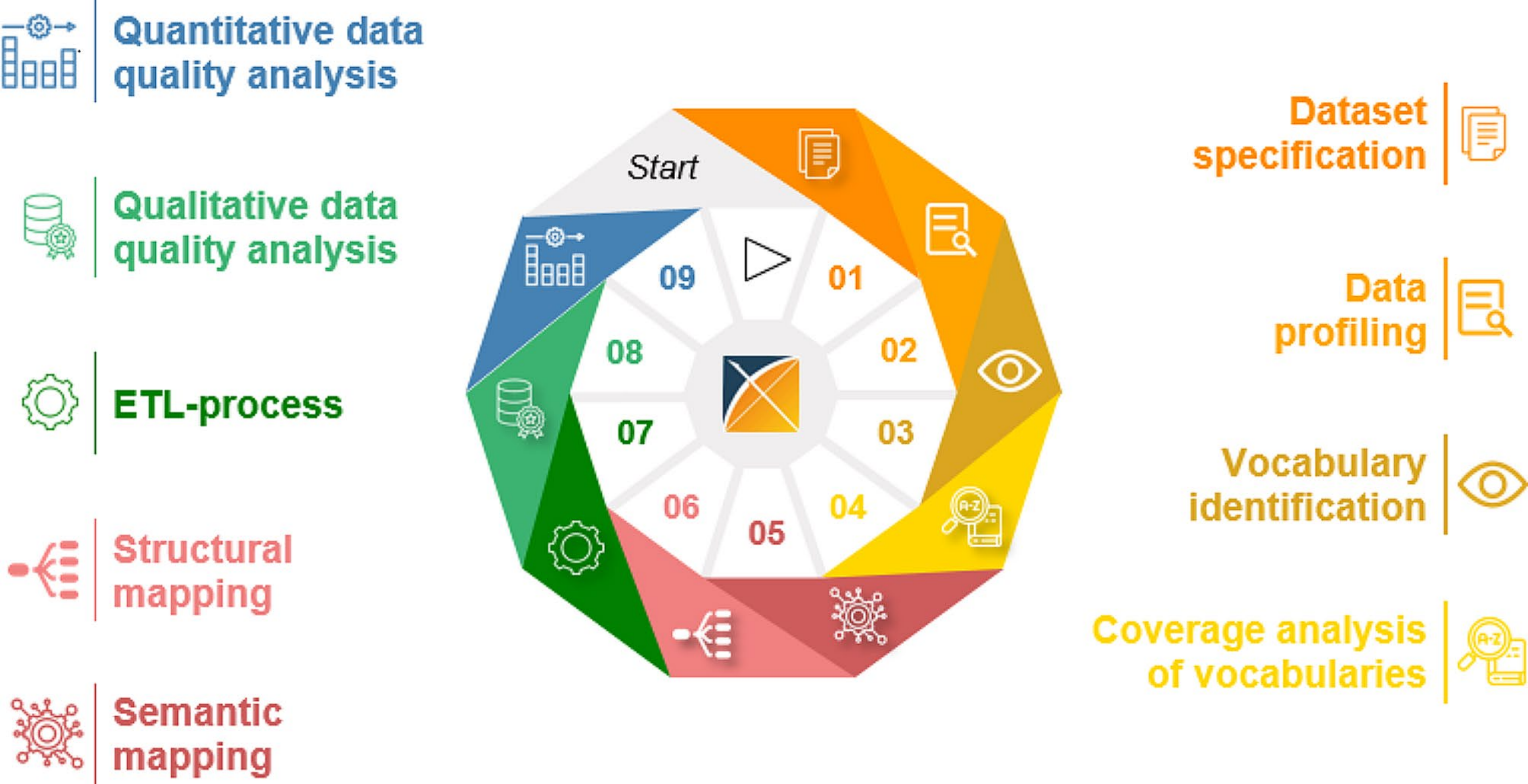
Generic data harmonization process for OMOP CDM; icons: Flaticon.com

Finally, to conceptualize a generic process for data harmonization in OMOP CDM, the sequence of process steps that showed the most agreement was determined. Figure 6 represents the resulting chronological order for the generic data harmonization process for OMOP CDM.

The generic data harmonization process consists of nine process steps in the following order:

1. Dataset specification.
2. Data profiling.
3. Vocabulary identification.
4. Coverage analysis of vocabularies.
5. Semantic mapping.
6. Structural mapping.
7. ETL-process.
8. Qualitative data quality analysis.
9. Quantitative data quality analysis.

## Discussion

The results show that we have achieved our goal of conceptualizing a generic data harmonization process for OMOP CDM. By conducting a literature review, we were able to answer our first research question. The literature review revealed nine process steps that were used by other researchers to harmonize clinical data and/or claims data in OMOP CDM. However, the results per publication show that not all steps have to be relevant. For example, Hong et al. [34], Hripcsak et al. [19], Lamer et al. [18], Lenert et al. [36] and Michael et al. [33] used only five from nine process steps. These five process steps also varied, as can be seen in the work of Hripcsak et al. [19] (dataset specification, vocabulary identification, semantic mapping, structural mapping, ETL-process) and Lenert et al. [36] (semantic mapping, structural mapping, ETL-process, quantitative and qualitative data quality analysis). Only the three process steps of semantic mapping, structural mapping and ETL-process are mentioned in all of the publications and thus form the mandatory part of the data harmonization process. Consequently, it is recommended to check individually for each data harmonization project whether process steps of the generic data harmonization process can be skipped if applicable.

Regarding our second research question, we identified seven OHDSI tools from the literature which were used to support the harmonization of clinical data or claims data in OMOP CDM. Overall, the results of the literature revealed that the OHDSI tools were not widely applied by researchers. This demonstrates that the use of OHDSI tools seems to be not mandatory.

Finally, our third research question was also addressed by looking at the chronological order of the identified process steps. A comparison of the results of the sequences of the three groups resulted in three different chronological orders of the nine process steps. This demonstrates that process steps can be interchanged if necessary while still achieving harmonization of source data in OMOP CDM (e.g. qualitative and quantitative data quality analysis). However, the interchange of structural mapping and semantic mapping for clinical data was surprising. Our experience during the harmonization of clinical data from FHIR format to OMOP CDM showed that in the majority of cases, it is not possible to do the structural mapping before the semantic mapping. The semantic mapping of source codes of free texts to concepts in the standardized vocabulary of OMOP CDM involves the assignment to a domain (e.g. Condition or Procedure). Domains specify the tables and fields in which source data should be transformed. Consequently, without this information a structural mapping was not possible. Nevertheless, there are some cases were an interchange of both process steps is plausible (e.g. exclusive use of demographic patient data).Despite the three different sequences, we were still able to derive a generic sequence of the nine process steps. Furthermore, it is important to mention that the generic data harmonization process should be considered as an iterative process. The last two steps of the qualitative and quantitative data quality analysis evaluate the correctness of the previous steps. If errors are identified, it may be necessary to perform the process again. In this context, it has to be checked individually whether all nine process steps have to be performed again or whether certain process steps do not contribute to the solution of the identified errors and can be skipped.

The present work is limited since only the harmonization of clinical data and claims data in OMOP CDM was focused. The literature review showed that there are also other types of data sources which are harmonized in OMOP CDM (e.g. registry data [47, 48]). In the future, it is therefore necessary to check whether the addition of other types of source data has an impact on the amount and sequence of the process steps. Furthermore, there are limitations in the interpretation of the results from group b) (claims data). The reason for this is that only a small number of the included publications used claims data (5/23 publications), which limits a comparison with clinical data (20/23 publications) and a subsequent generalization of the results.

A second limitation relates to the CDM used. Our work was focused on the OMOP CDM. However, there are many other CDMs, such as Sentinel [49], Informatics for Integrating Biology & Bedside (i2b2) [50] or the National Patient-Centered Clinical Research Network (PCORnet) [51] that harmonize source data in a standardized format. We believe that our work is also relevant and useful for researchers using other CDMs as a target format. The extent to which the 9-step process for OMOP CDM can be applied to other CDMs would need to be evaluated as future work.

Notwithstanding the limitations listed above, our generic data harmonization process provides a major benefit. Compared to OHDSI’s recommended 4-step process, our conceptualized 9-step process is more detailed. An analysis showed that our 9-step process can be assigned to the 4-step process of OHDSI as follows:

- OHDSI 1: Design the ETL.
  1. Dataset specification
  2. Data profiling
  3. Vocabulary identification
  4. Structural mapping

- OHDSI 2: Create the Code Mappings.
  4: Coverage analysis of vocabularies
  5: Semantic mapping

- OHDSI 3: Implement the ETL.
  7: ETL-process

- OHDSI 4: Quality Control.
  8: Qualitative data quality analysis
  9: Quantitative data quality analysis

The assignment demonstrated that all nine process steps extracted from the literature can be related to the OHDSI steps. However, we identified a difference in the chronological order of the nine assigned process steps. According to the sequence of the OHDSI steps, the structural mapping again would appear before the coverage analysis of vocabularies and the semantic mapping. The reason for this inconsistent order from OHDSI is that their process description [10] is focused on the use of the OHDSI tools (e.g. joint use of WhiteRabbit, RabbitInAHat) for data harmonization in OMOP CDM. In contrast, our process can be carried out independently of the OHDSI tools and used as a guide for other researchers to follow.

## Conclusions

Based on a literature review, necessary process steps for harmonizing clinical data or claims data in OMOP CDM were identified and placed in a chronological order. From these findings, a generic data harmonization process was derived. This process can be used as a step-by-step guide to assist other researchers in harmonizing source data in OMOP CDM. As future work, the applicability of the generic data harmonization process and OHDSI tools to German claims data will be investigated in practice. In this context, an evaluation will show whether further additional process steps need to be considered and to what extent the derived sequence is feasible in practice. Additionally, we plan to expand the guide in future to include best practices for the practical implementation and overcoming of challenges per process step and recommendations for using the OHDSI tools in this context.

### Electronic supplementary material

Below is the link to the electronic supplementary material.

A detailed overview of the results of the literature search, including the data extraction matrix can be found in the Additional file 1.

## Data Availability

No datasets were generated or analysed during the current study.

## Abbreviations

Achilles: Automated Characterization of Health Information at Large-scale Longitudinal Evidence Systems
BMBF: German Federal Ministry for Education and Research
CDM: Common Data Model
DQD: Data Quality Dashboard
ETL: Extract-Transform-Load
FHIR: Fast Healthcare Interoperability Resources
i2b2: Informatics for Integrating Biology & Bedside
MII: Medical Informatics Initiative
OHDSI: Observational Health Data Sciences and Informatics
OMOP: Observational Medical Outcomes Partnership
PCORnet: National Patient-Centered Clinical Research Network
PRISMA: Preferred Reporting Items for Systematic reviews and Meta-Analyses
SNOMED-CT: Systematized Nomenclature of Human and Veterinary Medicine Clinical Terms
